# Role of circulating endothelial cells in assessing the severity of systemic sclerosis and predicting its clinical worsening

**DOI:** 10.1038/s41598-020-80604-7

**Published:** 2021-01-29

**Authors:** Maria Luisa Di Martino, Alessandra Frau, Francesca Losa, Emma Muggianu, Mario Nicola Mura, Gianluca Rotta, Lorenza Scotti, Francesco Marongiu

**Affiliations:** 1 Department of Medical Sciences and Public Health and Unit of Internal Medicine, University of Cagliari, SS554-km 4,500, 09042 Monserrato, Cagliari Italy; 2 Becton Dickinson Biosciences Italy, Milan, Italy; 3 Department of Translational Medicine, University of Piemonte Orientale, Novara, Italy

**Keywords:** Immunology, Biomarkers, Diseases, Rheumatology, Risk factors

## Abstract

Endothelial damage and fibro-proliferative vasculopathy of small vessels are pathological hallmarks of systemic sclerosis (SSc). The consequence is the detachment of resident elements that become circulating endothelial cells (CECs). The aim of our study was to evaluate the potential of CECs as biomarker in SSc. We enrolled 50 patients with limited cutaneous (lcSSc) and diffuse cutaneous (dcSSc) subset of SSc, who underwent clinical evaluation to establish the organ involvement. CECs were measured by flow-cytometry utilizing a polychromatic panel. An evident difference was observed in CEC counts comparing controls to SSc patients (median 10.5 vs. 152 cells/ml, *p* < 0.0001) and for the first time, between the two subsets of disease (median lcSSc 132 vs. dcSSc 716 CEC/ml, *p* < 0.0001). A significant correlation was established between CECs and some SSc clinical parameters, such as digital ulcers, skin and pulmonary involvement, presence of Scl-70 antibodies, nailfold videocapillaroscopy patterns and EUSTAR activity index. After 12 months, CECs correlated with clinical worsening of patients, showing that a number higher than 414 CEC/ml is a strong negative prognostic factor (RR 5.70). Our results indicate that CECs are a direct indicator of systemic vascular damage. Therefore, they can be used as a reliable marker of disease severity.

## Introduction

Systemic sclerosis (SSc) is an autoimmune disease characterized by fibro-proliferative vasculopathy of small vessels. It is associated with immunological dysregulation paralleled by excessive collagen and matrix components deposition in the skin and internal organs^1^. It has a prevalence of 1–5/10,000 and female:male sex ratio of about 6:1^2,3^. Typically patients are classified in three different subsets: limited cutaneous SSc (lcSSc), diffuse cutaneous SSc (dcSSc), and SSc without skin involvement (sine scleroderma SSc). In lcSSc patients skin involvement affects extremities, but can involve the face and neck; in dcSSc skin involvement extends proximally to the elbows and knees; in patients with sine scleroderma SSc are found internal organ involvement and serological abnormalities in absence of skin thickening^2,4^. The clinical hallmarks of the disease, such as Raynaud phenomenon and digital ulcers, are the consequence of peripheral vascular damage, which occurs early in the pathogenesis. Indeed, endothelial disruption and vascular reactivity precede by several years the fibrosis onset. Endothelial cells damage leading to cells detachment from vessel walls and subsequent apoptosis, involves primary small arteries, arterioles and capillaries. Hence, disease progression and the institution of the ischemia–reperfusion process promote vascular remodeling, with intima and media hypertrophy, adventitia fibrosis and progressive lumen occlusion. In parallel, reduced blood flow and chronic tissue hypoxia drive a vicious circle that, together with impaired angiogenesis and vasculogenesis, produce severe organ damage, such as pulmonary arterial hypertension (PAH) and scleroderma renal crisis^5,6^. Combined, the evidence lead researchers to focus on the role of endothelial cells as possible candidates of disease biomarkers^7^.

The loss of vessel wall integrity consequentially leads to the detachment of elements that become circulating endothelial cells (CECs). CECs are characterized by a mature endothelial phenotype and represent between 0.01 and 0.0001% of mononuclear cells in normal peripheral blood^8^. The pathophysiological significance of these cells was studied in many conditions, from cancer to cardiovascular disease and systemic vasculitis^9–12^. Literature suggests that CECs tend to increase as a consequence of endothelial damage, offering to clinicians a potentially promising biomarker of vascular impairment. The rarity of CECs in the bloodstream requires a reliable method for their identification and polychromatic flow cytometry (PFC) is considered the technique of choice^13^. Nevertheless, the use of different approaches has produced discordant results among research groups^14–16^, when CECs have been evaluated in SSc patients^17,18^. By taking advantage of a new PFC panel that has recently been standardized in healthy subjects through a multicenter study^19^, we have evaluated CEC counts in SSc patients. In this panel, CECs are defined as live/nucleated/CD45negative/CD146positive/CD34bright elements^19,20^.

The goal of our study was to assess the correlation between CEC counts, disease severity and progression. We aimed to candidate CEC as a new reliable biomarker for patient stratification and disease worsening.

## Results

### Demographic and clinical features of patients

The present study included 46 woman and 4 men, with a mean age of 62 years (range 33–77 years); the mean disease duration was 10 years (range 2–26 years). According to 2013 ACR-EULAR classification criteria^4^, patients with skin involvement were stratified into two subsets of disease, the limited cutaneous (33 subjects) and the diffuse cutaneous form (17 subjects), nobody presented SSc sine scleroderma subset.

40 healthy donors, matched by age and sex were also included as control group.

The clinical characteristics of patients are summarized in Table 3.

### Evaluation of CECs

**Figure 1:**
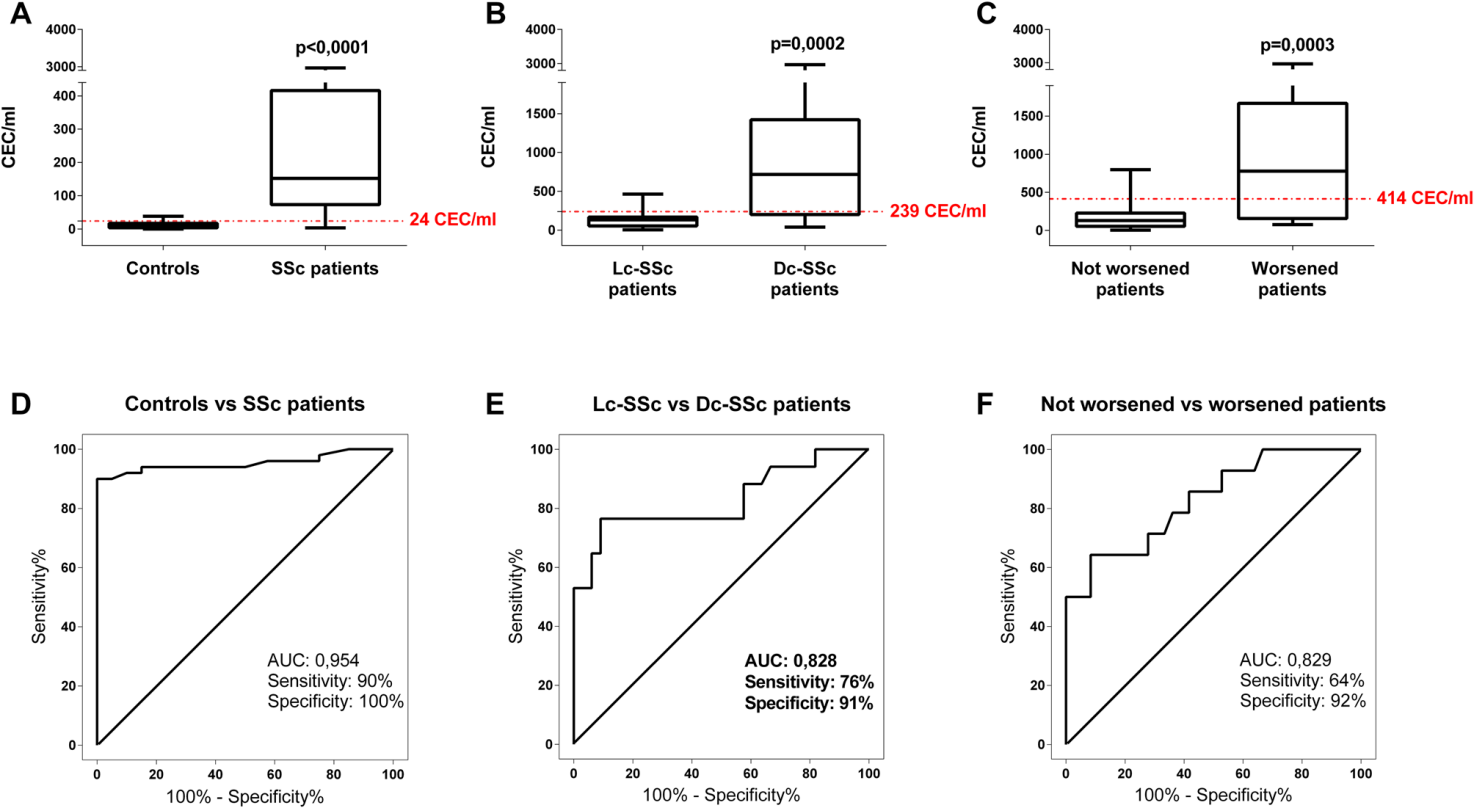
Circulating endothelial cells (CEC) in healthy controls and SSc patients. Boxplots show the median CEC counts and whiskers from minimum to maximum comparing healthy donors and SSC patients (**A**), limited cutaneous (lc-SSc) and diffuse cutaneous (dc-SSc) patients (**B**), not worsened versus worsened patients (**C**). Red grid line in boxplots indicates the CEC value cut-off of related ROC curve. D, E, F represent ROC-curves of the absolute CEC count distinguishing controls from SSc patients (**D**), limited cutaneous (lc-SSc) and diffuse cutaneous (dc-SSc) patients (**E**), not worsened from worsened SSc patients (**F**).

The median CEC value in SSc patients was higher in comparison to healthy subjects (152 cells/ml, IQR 76–414 vs. 10.5 cells/ml, IQR 4–16.5; *p* < 0.0001). The cut-off value that best discriminated between controls and patients was 24 cells/ml (Fig. 1A), showing 100% of specificity and 90% of sensitivity (AUC 0.954; Fig. 1D). Moreover, the CEC counts strictly correlated with the different subsets of disease (*p* = 0.0002), showing a median of 716 cells/ml (IQR 243–1334 cell/ml) for the dcSSc versus a median of 132 cells/ml (IQR 55–167) for the lcSSc group. The cut-off value, that best discriminated between the limited and diffuse groups was 239 cells/ml, (Fig. 1B) with 76% of sensitivity and 91% of specificity (AUC 0.828, Fig. 1E). Our results also indicated that CECs counts can discriminate between worsening and not worsening patients (*p* = 0.0003, Fig. 1C), being the best cut-off 414 CEC/ml with a 92% of specificity and a 64% of sensitivity (AUC 0.829, Fig. 1F).

In addition, CEC count strictly correlated with most of the relevant clinical parameters assessed at SSc diagnosis and follow up (Table 1).

**Table 1:** Quantification of CECs/ml in controls and patients and association between the two subsets of disease and the clinical features.

	First evaluation			Second evaluation		
	CEC median (cells/ml)	CEC range (IQR)	*p*-value	CEC median (cells/ml)	CEC range (IQR)	*p*-value
**Subjects**						
Healthy subjects	10.5	4–16.5	** < 0.0001**	–	–	< 0.0001*
SSc patients	152	76–414		158	50–419	
**Subset of SSc**						
Limited cutaneous SSc patients	132	55–167	**0.0002**	87	36–165	** < 0.0001**
Diffuse cutaneous SSc patients	716	243–1334		518	292–1741	
**Clinical features of patients**						
Digital ulcers						
Absence	128	56–262	**0.0100**	124	37–335	**0.0093**
Presence	286	155–1136		261	166–2080	
**mRSS**						
< 18	122	44–258	**0.0005**	75	20–167	**0.0001**
18–30	286	105–1136		237	110–998	
> 30	830	235–2321		2270	356–4382	
**HRCT**						
Normality	37	18–137	**0.0018**	22.5	5–42	**0.0020**
Early septal interlobular fibrosis	132	62–250		100	38–231	
Ground glass	169	145–908		200	114–465	
Honeycombing	759	86–1632		723.5	309–1442	
**DLCO**						
Normal (76–140%)	99.5	37–193.5	**0.0226**	73.5	25–180	**0.0094**
Reduction (≤ 75%)	171	125–442.5		203.5	116.5–448.5	
**Ab anti-SCL70**						
Absence	136	58–207	**0.0024**	Not repeated		
Presence	716	107–1334				
**Ab anti-ACA**						
Absence	241	38–1104	0.5371	Not repeated		
Presence	136	83–170				
**Capillaroscopic patterns** (**NVC**)						
Unspecific alteration	Not available			24	12–146	**0.0002**
Early scleroderma pattern				43	4–216	
Active scleroderma pattern				156	44–213	
Late scleroderma pattern				356	110–1075	
**Revised EUSTAR activity index**						
Inactive/moderately active < 2.5	Not available			63	15–168	**0.0015**
Active/very active ≥ 2.5				237	123–482	
**Aesophageal dismotility**						
Normal	148	130–288	0.5712	69	12–249	0.0744
Pathological	146	64–414		160	61–448	
**PAPs**						
≤ 40 mmHg	162	80–416	0.2859	154.5	47–433.5	0.4594
> 40 mmHg	76	46–407		57	–	

Median CEC value/ml in peripheral blood, interquartile range (IQR) of values and overall *p*-value during first and second evaluation. The association between CECs, revised EUSTAR activity index and NVC were calculated only during the second clinical evaluation of patients. Data highlighted in bold indicate statistically significant association.

*Comparison with values of healthy subjects at first evaluation.

A significant increase was observed in patients affected by digital ulcers when compared with matched ulcer free patients (286 CEC/ml vs. 128 CEC/ml, *p* = 0.01). Moreover, although no clear difference was observed comparing patients with mRSS between 18 and 30 and patients with mRSS > 30, a strong and significant correlation emerged comparing patients with mRSS > 30 to subjects with mRSS < 18 (830 CEC/ml vs. 122 CEC/ml, *p* = 0.0005).

CEC counts highly correlated also with pulmonary fibrosis (*p* = 0.0018). Patients with ground glass (169CEC/ml) and honeycombing (759 CEC/ml) anomalies showed higher CEC number when compared to those with early septal interlobular fibrosis (132 CEC/ml) and those with a normal pulmonary pattern (37 CEC/ml). In parallel, CEC number was significantly higher (*p* = 0.0226) in patients with reduction in DLCO (171 CEC/ml) respect to patients showing normal DLCO (99.5 CEC/ml).

Concerning the laboratory data, CECs showed a significant difference between the patients with or without anti-SCL70 antibodies (716 vs. 136 CEC/ml, *p* = 0.0024). No statistical correlation was found between CECs, ACA, PAPs and aesophageal dismotility. After twelve months, CEC patterns were found stable in the overall population (geometric mean 156 vs. 142 CEC/ml). Interestingly, the 14 patients undergoing disease worsening according to one or more clinical parameter, highlighted during the first evaluation, a clear CEC count difference with respect of the not-worsening counterpart (776 vs. 130 CEC/ml *p* = 0.0003). During the second evaluation, we performed nailfold videocapillaroscopy (NVC), patients with late scleroderma pattern showed highest CEC value (356 CEC/ml) in respect of those with unspecific alterations (24 CEC/ml), early (43 CEC/ml) and active (156 CEC/ml) scleroderma pattern (*p* = 0.0002). Moreover, patients with active/very active disease, determined according to EUSTAR Activity Index showed a higher CEC value than those with inactive/moderately active disease (237 vs. 63 CEC/ml; *p* = 0.0015).

The results of the univariate models are reported in Table 2.

**Table 2:** Relative risk (RR) and corresponding 95% Confidence intervals for the significant association between patient characteristics and disease worsening.

Variable	RR	95% CI
**Subset of SSc**		
Diffuse cutaneous versus limited cutaneous	3.49	(1.39–8.80)
**mRSS**		
18–30 versus < 18	3.41	(1.21–9.65)
> 30 versus < 30	3.63	(0.95–13.81)
**Ab anti-SCL70**		
Presence versus absence	2.85	(1.23–6.56)
**CEC**		
> 414 versus ≤ 414	5.70	(2.37–13.74)

Patients with diffuse form of the disease have an increased risk of worsening compared to patients with limited form, as well as those with mRSS values between 18 and 30 and with anti-SCL70 antibodies. Finally, patients with CEC values greater than 414 have a strong increased risk of worsening.

## Discussion

The discovery of new biomarkers in connective tissue diseases, efficient in predicting clinical outcome and/or therapy response, is a challenging purpose in the field of clinical research. To date, the role of serum autoantibodies is well established in diagnosis and classification of systemic sclerosis (SSc)^21^, yet, in spite of their high specificity, the lack of sensitivity in detecting major organ complications during patient follow-up make them unsatisfactory prognostic markers^22^. Indeed, different molecules released during vascular damage and tissue fibrosis have been investigated thoroughly as potential biomarkers^23^. Clinicians agree to consider vasculopathy as the earliest pathogenic mechanism in the onset of SSc, throughout endothelial damage, impaired angiogenesis and vasculogenesis^1^, nevertheless these mechanisms are difficult to measure by means of in vitro standard approaches.

The aim of our study was to assess the clinical relevance of CEC evaluation in SSc patients, as direct biomarker of systemic endothelial damage, to be used in patient stratification and disease activity/progression monitoring. The role of CECs in SSc had already been investigated, however the results obtained were discordant and incomparable, likely due to different technical approaches^17,18,24^. Our data, generated by a highly standardized flow cytometry method, confirmed the rarity of CECs in healthy subjects in comparison to SSc patients and, for the first time, highlighted a clear difference between the limited cutaneous (lcSSc) and the diffuse cutaneous (dcSSc) form. This significant difference in CEC count is fully consistent with the extent of vascular damage, being this definitively greater in patients affected by dcSSc. In fact, despite sharing the same pathogenic mechanism, they normally show different clinical course and prognosis. In addition, we observed a number of strict correlations between CEC values and clinical, instrumental and laboratory parameters routinely used in clinical practice. The number of CECs was higher in patients affected by active digital ulcers and showing high mRSS value. The presence of lung fibrosis, assessed either by both HRCT and spirometry with DLCO analysis, showed a tight correlation with CECs, in particular in the dcSSc, where lung involvement is early and more evolutive^25^. Additionally, the presence of high value of CECs in patients displaying anti-SCL70 Ab represents a further proof of capacity of this assay to discriminate patients with dcSSc^26^. The occurrence of pulmonary hypertension (PAH) did not correlate with CEC counts, even though endothelial dysfunction plays a prominent role in the developing of PAH, which tends to be more serious and early, especially in lcSSc^27^. These results may be explained considering that the trans-thoracic echocardiography (TTE), a screening tool for PAH, has remarkable measurement variability and may not be enough sensitive for the detection of early disease^28^. On the other hand, to perform cardiac catheterism did not appear to be ethical. After twelve months from the first assessment, CEC evaluation was repeated, as part of patient follow-up, confirming the other clinical and instrumental parameters with the evaluation of nailfold videocapillaroscopy (NVC) and revised EUSTAR activity index.

Capillaroscopic examination, utilized to evaluate the microcirculation in vivo, can well differentiate between isolated Raynaud’s phenomenon, and early SSc abnormalities, even if SSc NVC pattern can also be seen in other connective tissue disease^29^. CEC counts represent an interesting companion test to NVC being capable to provide specific information related to systemic endothelial damage, that reinforce and corroborate the local morphological assessment performed at the nail fold. Consistently, a strong correlation between CEC numbers and the NVC was detected, in particular with late and active patterns.

The observation that CECs strongly correlated with the revised EUSTAR activity index, as well as with several clinical parameters assessing organ dysfunction, suggest their potential as systemic endothelial damage biomarker.

In our cohort, the value of CECs remained almost stable after one year, suggesting that the method is reproducible. Indeed, patients with stable disease showed similar cells counts in the two evaluations; on the contrary, CECs were increased in patients with a progressive disease. Interestingly, we also found that CEC numbers higher than 414 cells/ml were a strong negative prognostic factor, indicating the risk of disease aggravation (RR 5.70), definitely stronger than others clinical and instrumental parameters (Table 2). Furthermore, in comparison with auto-antibodies evaluation (anti-Scl-70, anti-ACA and anti-RNA polymerase III) CEC counts attested a much higher sensibility and similar specificity^21^. Taken together, our results demonstrate that CECs represent a sensitive tool to distinguish healthy subjects from patients, and for the first time, they are shown as a robust, reproducible biomarker capable to distinguish the different subsets of SSc and to assess disease severity and progression.

CEC enumeration, together with other endothelial dysfunction tests should be considered as a valuable approach to better understand the role of endothelial damage/dysfunction in the onset and progression of systemic sclerosis, especially to fully examine whether the two subsets of disease truly share the same pathogenic mechanism and differ only in the district/extension of damage. At present, is still to be clarified whether the number of CECs can precede the disease clinical manifestations. Accordingly, the CEC quantification in “early” and “very early” scleroderma patients, may be an interesting test to help early diagnosis and could provide information about their possible role as a predictive factor. Furthermore, it will be necessary to verify whether pronounced increase of CECs is a peculiarity of SSc patients only, or if the same vascular damage can be associated with other connectivities and/or autoimmune disease.

Our study has also some limitations that should be considered. First, a small group of patients has been analyzed, however, the disease is rare and results are strong enough to draw solid conclusions. Second, all patients were treated with different drugs, as most of them present a long duration of disease; this could have affected the course of the disease and thus the evaluation of CEC behavior.

Thirdly, the low number of patients who worsened their disease did not allow to verify if CEC were independently associated to patients’ worsening.

Bridging the gap, it is in our future plans to estimate CECs at SSc diagnosis, before initiating the therapy and after any treatment change, to verify how this may be predictive and prognostic of future disease development.

In conclusion, our data demonstrated that CEC enumeration is a valuable and reproducible test to assess severity and progression of SSc, able to support the clinicians in the diagnosis and patient follow-up.

## Methods

### Subjects

The present study was approved by local ethical committee. A total of 50 SSc patients and 40 healthy donors were enrolled in the present study after written informed consent. Patients were recruited according to the 2013 ACR/EULAR criteria^4^. All patients received monthly therapy with intravenous Iloprost, a synthetic analogue of prostacyclin PGI2^30,31^. They were also in therapy with vasodilators such as calcium antagonists, ACE inhibitors or antagonists of the endothelin receptor and with immunosuppressive drugs such as methotrexate, azathioprine, mycophenolate mofetil and prednisone at doses lower than 10 mg, according to the organ involvement. The exclusion criteria for patients and controls were the cigarette smoke, acute infectious states, active gastric or duodenal ulcer, positive serology for HIV virus and autoimmune or neoplastic diseases.

### Clinical assessment

Clinical, laboratory and instrumental evaluations were carried out in each patient within one month before and maximum two months after the CEC counts. The same cohort of patients underwent a second evaluation of CEC counts in addition to clinical and instrumental assessment after twelve months, as follow-up (Table 3).

**Table 3:** Demographic and clinical characteristics of patients.

	First evaluation	Second evaluation
**SSc patients**	50	50
Males	4 (8%)	4 (8%)
Females	46 (92%)	46 (92%)
Mean age (range)	62 years (33–77)	63 years (34–78)
Mean SSc duration (range)	10 years (2–26)	11 years (3–27)
**Healthy controls**	40	
Males	6 (15%)	
Females	34 (85%)	
Mean age (range)	57 (27–72)	
**Clinical features of patients**		
**Subset of SSc**		
Limited cutaneous	33 (66%)	33 (66%)
Diffuse cutaneous	17 (34%)	17 (34%)
Sine scleroderma	0 (0%)	0 (0%)
**Raynaud phenomenon**		
Absence	1 (2%)	8 (16%)
Presence	49 (98%)	42 (84%)
**Digital ulcers**		
Absence	37 (74%)	38 (76%)
Presence	13 (26%)	12 (24%)
**mRSS**		
< 18	29 (58%)	24 (48%)
18–30	17 (34%)	23 (46%)
> 30	4 (8%)	3 (6%)
**HRCT**		
Normality	6 (12%)	4 (8%)
Early septal interlobular fibrosis	25 (50%)	22 (44%)
Ground glass	13(26%)	10 (20%)
Honeycombing	6 (12%)	8 (16%)
Missing data	0	6 (12%)
**DLCO**		
Normal (76–140%)	20 (40%)	16 (32%)
Reduction (≤ 75%)	27 (54%)	28 (56%)
Missing data	3 (6%)	6 (12%)
**PAPs**		
≤ 40mHg	45 (90%)	44 (88%)
> 40 mmHg	5 (10%)	3 (6%)
Missing data	0	3 (6%)
**Aesophageal dismotility**		
Absence	7 (14%)	8 (16%)
Presence	42 (84%)	40 (80%)
Missing data	1 (2%)	2 (4%)
**Autoantibody pattern**		
ANA (titre ≥ 1:160)	47 (94%)	Not repeated
Anti-SCL70 positive	13 (26%)	Not repeated
Anti-centromere (ACA) positive	24 (48%)	Not repeated
**Capillaroscopic patterns** (**NVC**)		
Unspecific alteration		6 (12%)
Early scleroderma pattern		6 (12%)
Active scleroderma pattern		15 (30%)
Late scleroderma pattern		23 (46%)
**Revised EUSTAR activity index**		
< 2.5		17 (34%)
≥ 2.5		27 (54%)
Missing data		6 (12%)

Epidemiological and clinical features of enrolled subjects are expressed as absolute numbers and percentages. The nailfold videocapillaroscopy (NVC) and revised EUSTAR activity index were included in the second evaluation only.

By the clinical point of view, the duration and frequency of the Raynaud phenomenon and the presence of ulcers were evaluated. The skin involvement was estimated using the modified Rodnan skin score (mRSS)^32^ and choosing the values < 18, 18–30 and > 30 to separate patients into three classes of severity for cutaneous thickening^33^. Pulmonary involvement was studied by thoracic high-resolution computed tomography (HRCT). Lung fibrosis, was classified into four groups, starting from normality, early septal interlobular fibrosis, presence of ground-glass pattern, up to honeycombing pattern. In order to evaluate gas exchange capacity for the presence of lung interstitial disease, patients were stratified into 2 groups according to the value of the carbon monoxide diffusing capacity (DLCO), considering as pathological a percentage lower than 76%^25^. An estimate of pulmonary artery systolic pressure (PAPs) has been calculated through color Doppler echocardiography, considering pathologic values higher than 40 mmHg at rest. The presence of aesophageal dismotility was assessed by radionuclide aesophageal transit time^34^. Anti-topoisomerase I (Scl-70) and anti-centromere (ACA) specific antibodies was detected using the enzyme-linked immunosorbent assay (ELISA) and indirect immunofluorescence (IFI), respectively^21^.

Unfortunately, only during the second clinical evaluation all patients underwent nailfold videocapillaroscopy (NVC) and the evaluation of EUSTAR activity index. The qualitative assessment of capillaroscopic features was reported according to the NVC pattern by Cutolo et al*.*: early, active and late^35^. Patients whose capillaroscopic characteristics did not meet any of the three patterns were classified as subjects with "unspecific alterations". Moreover, the disease activity was assessed using the revised EUSTAR activity index by Valentini et al*.*^33^. A value ≥ 2.5, obtained by the sum of the scores, has been chosen as cut-off to distinguish between active/very active from inactive/moderately active disease.

### Sample collection and flow cytometry CEC analysis

**Figure 2:**
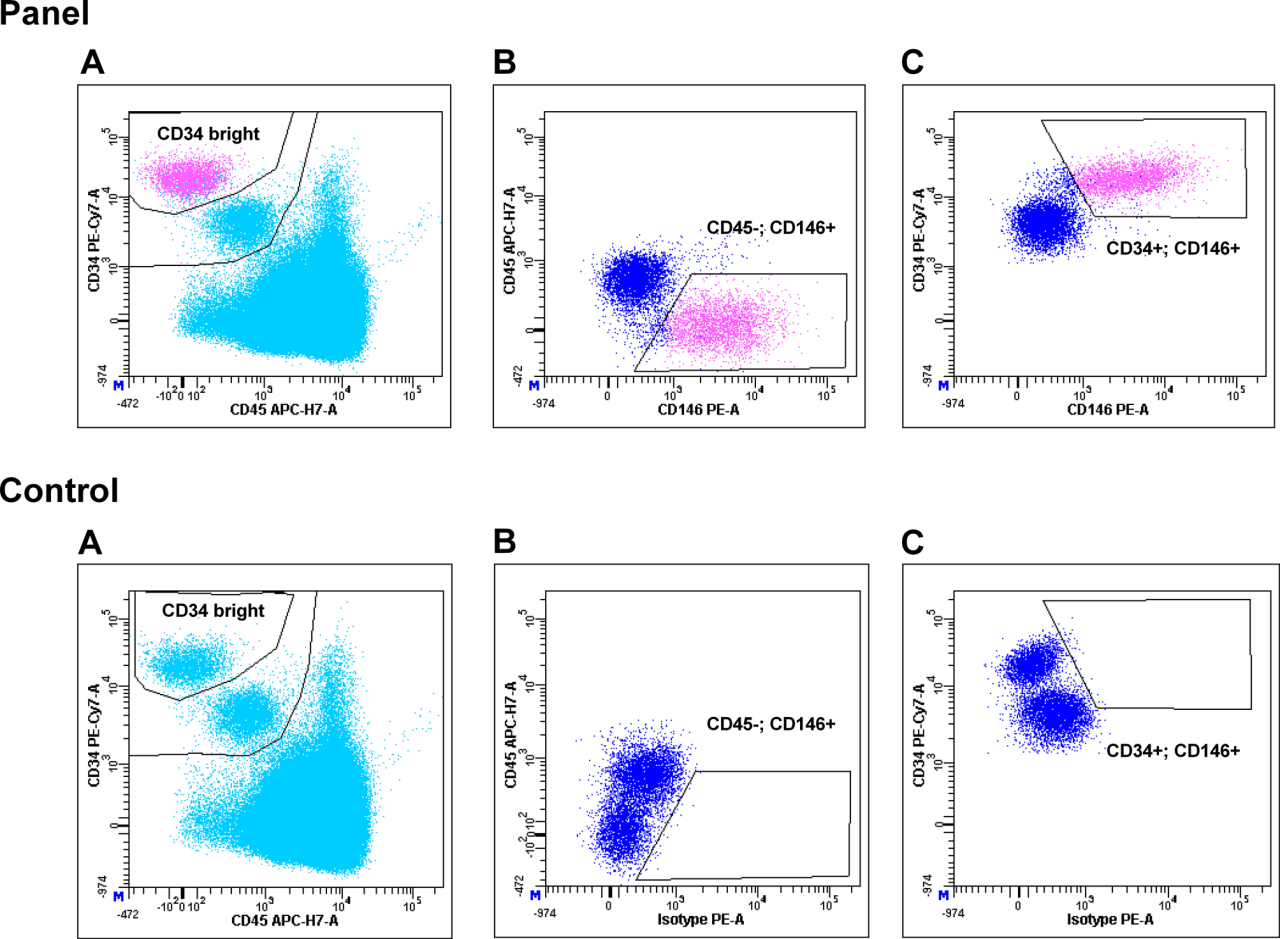
Flow cytometry characterization of Circulating Endothelial Cells (CEC). Live nucleated lympho-monocytes were assessed for CD45 and CD34 expression (sky-blue dots in plots A, panel and control). The population defined as positive for CD34 and negative for CD45 expression was gated (blue dots), and evaluated for CD146 positivity. CD146 was gated either versus CD45 (plot B panel) and versus CD34 (plot C panel). Real CD34 bright, CD146 positive events (pink dots) resulted from the intersection of gates displayed in plot A, B and C are CECs. The position of these gates was defined on the basis of matched isotype controls (plot B and C control).

Peripheral blood samples were collected immediately before Iloprost infusion, and at least 1 month after previous administration. Each sample was collected in K2E EDTA tubes and processed within 4 h from bleeding. The first drawn tube was used to determine leukocyte absolute count, in order to enumerate CEC by dual-platform counting procedure. Samples were processed according to the method described by Lanuti et al.^20^ Briefly, a volume of peripheral blood containing 20 × 10^6^ leukocytes underwent an erythrocyte-lysis step, with 1X Pharm Lyse solution (BD Biosciences) according to manufacturer’s instructions. The washed cellular pellet was resuspended and added to Circulating Endothelial Cells Lyotube (BD Biosciences), containing a lyophilized cocktail of: 7-AAD, CD45 APC-H7; CD34 PE-Cy7; CD146 PE; CD309 AlexaFluor647. 50 µl of Syto16 (Invitrogen, USA) 1:1000 was added as liquid drop in. Samples were then incubated for 30′ at 4 °C, washed (2 ml of Stain Buffer with BSA, BD Biosciences) and re-suspended in FACSFlow (BD Biosciences). 3,1 × 10^6^ events/sample with lympho-monocyte morphology (SSC/FSC dot plot) were acquired by flow cytometry (FACSCanto II, standard configurations) and analyzed using BD FACSDiva v 8. Specific analysis gate strategy was used to enumerate live CECs, defined as negative for 7-AAD and CD45, positive for Syto16, CD146 and bright for CD34. Control tube containing isotype control for CD146 PE was used to define the positivity region for this marker (Fig. 2). CEC counts were always expressed as number of cells/ml of peripheral blood. Instrument performances and data reproducibility were checked according to manufacturer’s instructions, before each evaluation.

### Statistical analysis

Results are presented as median and interquartile range (IQR) being data not normally distributed. To evaluate if the CECs count differs between SSc patients and healthy controls, disease forms (limited cutaneous vs. diffuse cutaneous) and between worsened and not worsened patients, the Mann–Whitney test was used to test the median difference. Moreover, receiving operational curves (ROC) were calculated to identify the best value of CEC distinguishing between the SSc patients and controls, the lcSSc and dcSSc and clinical deterioration considering the data collected at the second evaluation. The best CEC number was identified and the corresponding sensitivity and specificity values calculated. The area under the curve (AUC) was used to assess the overall classification ability of the CECs. To assess the differences in the median CECs count between patients’ characteristics in the first and second evaluation, Wilcoxon sum rank and Kruskal Wallis analysis of variance were applied for categorical variables with two or more than two categories respectively. Finally, to evaluate which characteristics were associated to the disease worsening, univariate logistic binomial regression models were applied. Results are reported as Relative risk (RR) and corresponding 95% confidence intervals (95%CI). Results were considered statistically significant when the *p*-values were < 0.05. All analyses were performed using MedCalc statistical software. The graphical exploration was performed in Graph Pad v.7 software.

### Ethical approval

The independent Ethics Committee of A.O.U. of Cagliari (Italy) approved this study. All methods were performed in respect for life and the person as indicated in the Charters of Human Rights, in the Recommendations of international and national Organizations, in the national and international Medical Deontology and in particular in the current revision of the Helsinki Declaration, in the Convention on human rights and Biomedicine of the Council of Europe (Oviedo).
